# Botulinum Toxin Type A in Spasticity and Cervical Dystonia: Practical Clinical Recommendations from a Mexican Multidisciplinary Panel of Specialist Injectors

**DOI:** 10.3390/toxins18070287

**Published:** 2026-06-30

**Authors:** Jorge Hernández Franco, Salvador J. Santamaría Molina, José J. Zorrilla Sánchez, Jorge Carranza del Río, Israel Sánchez Villavicencio, Sofía D. Hernández, Emmanuel Duvignau Dondé, Héctor A. González Usigli, Yadira S. González López, Roberto Leal Ortega, Azael A. Flores Salinas, Laura A. Mejía Alonso, Juan F. J. Gómez Hernández, Moisés Fernández Bravo, Pedro I. Arias Vázquez

**Affiliations:** 1 National Institute of Neurology and Neurosurgery “Manuel Velasco Suárez” (INNN), Mexico City 14269, Mexico; isvi.rehabilita@gmail.com; 2 Mexican Social Security Institute (IMSS), National Medical Center La Raza, Mexico City 02990, Mexico; chavin_75@yahoo.com; 3 Teleton Children’s Rehabilitation and Inclusion Center (CRIT), Mexico City 09868, Mexico; zorrilla@teleton.org.mx (J.J.Z.S.); gonzalez@teleton-neza.org.mx (Y.S.G.L.); javier.gomez@teleton.org.mx (J.F.J.G.H.); 4 Association for People with Cerebral Palsy (APAC), Mexico City 06720, Mexico; jcarranza_mx@yahoo.com; 5 Institute for Social Security and Services for State Workers (ISSSTE), General Hospital Tacuba, Mexico City 11410, Mexico; dra.sofiaduranhernandez@gmail.com; 6 Pediatric Rehabilitation Center of the Armed Forces, Mérida 97196, Mexico; dr.duvignau@gmail.com; 7 Mexican Social Security Institute (IMSS), Western National Medical Center (CMNO), Guadalajara 44329, Mexico; quincy1973@hotmail.com; 8 Regional High Specialty Hospital of the Yucatán Peninsula (HRAEPY), Mérida 97130, Mexico; drleal13@hotmail.com; 9 PHYSIS High-Specialty Rehabilitation, Chihuahua 31203, Mexico; mrazaelflores@gmail.com; 10 Mexican Social Security Institute (IMSS), National Medical Center Siglo XXI, Mexico City 06720, Mexico; ale.rehabilita@gmail.com (L.A.M.A.); drmfb@hotmail.com (M.F.B.); 11 Academic Division of Health Sciences, Juárez Autonomous University of Tabasco (UJAT), Villahermosa 86040, Mexico; pivanav@gmail.com

**Keywords:** spasticity, cervical dystonia, botulinum toxin, botulinum toxin type A, best practices, neuromuscular disorders, clinical recommendations

## Abstract

Cervical dystonia and spasticity are debilitating neuromuscular disorders that substantially impair quality of life. Botulinum toxin type A (BoNT-A) is a cornerstone therapy; however, heterogeneity in dosing, muscle selection, and guidance techniques can limit outcomes, and Mexico-specific practical guidance is limited. We convened a multidisciplinary panel of Mexican rehabilitation and neurology specialists with extensive experience in BoNT-A injection; recommendations were developed through a three-phase process: a targeted literature search, a structured online survey of Mexican rehabilitation and neurology specialists, and an in-person meeting (21 February 2025) where clinicians reviewed aggregated survey results and refined practical recommendations through structured deliberation. The manuscript provides pragmatic guidance on evaluation and goal setting, muscle selection, working dilutions, dosing ranges, and reinjection intervals, emphasizing ultrasound (US) and/or electromyography (EMG) for deep or high-risk targets. It also summarizes key adverse events and proposes a stepwise approach to inadequate response, prioritizing reassessment of diagnosis, goals, targeting, and techniques before considering immunogenicity testing, product switching, or alternative interventions. These recommendations aim to improve standardization, safety, and goal attainment in routine Mexican practice.

## 1. Introduction

Spasticity and dystonia are neurological disorders that may occur at any age; both conditions can lead to difficulties in daily functioning and impair quality of life. Spasticity is characterized by a velocity-dependent increase in stretch-reflex activity, whereas cervical dystonia is marked by sustained or intermittent contractions of neck muscles that force the head into abnormal positions; botulinum toxin type A counteracts both forms of muscle overactivity by blocking neuromuscular transmission [1].

BoNT-A is widely recognized as the first-line treatment for focal and multifocal muscle overactivity, due to its favorable efficacy and safety profile compared with systemic pharmacological therapies [1]. The administration of BoNT-A is a complex and highly individualized therapeutic process, guided by structured clinical algorithms and patient-specific injection protocols. These algorithms provide a comprehensive framework encompassing key parameters such as muscle selection, dosing ranges, dilution ratios, guidance modalities, and injection intervals [2,3].

Despite multiple international initiatives aimed at standardizing the use of BoNT-A, existing guidelines vary considerably in their scope, methodological rigor, and clinical applicability. Most available recommendations have been developed in the United States and Europe, reflecting healthcare environments, regulatory systems, and clinical infrastructures. To date, there are no published clinical practice guidelines from Latin America—or specifically from Mexico—that address the region’s unique epidemiological, logistical, and educational considerations. This absence highlights the critical need for context-specific guidance [2,3,4,5,6,7].

The present work seeks to fill this gap by offering evidence-informed, practice-oriented recommendations based on the collective expertise of Mexican specialists in rehabilitation and neurology with extensive experience in BoNT-A therapy. These statements aim to support clinical decision-making and optimize therapeutic outcomes, ultimately enhancing patient quality of life. Recommendations are formally endorsed by the Colegio Mexicano de Medicina de Electrodiagnóstico, Medicina Física y Rehabilitación (Mexican College of Electrodiagnostic Medicine and Physical and Rehabilitation Medicine, COMEFYR), underscoring their clinical relevance and institutional legitimacy within the national healthcare context.

## 2. Results

Main practice statements are summarized in Table 1. It integrates key recommendations on clinical assessment, goal setting, timing of BoNT-A initiation, injection technique, dosing considerations, reinjection intervals, and practical dilution strategies for each therapeutic area.

### 2.1. Adult Spasticity

#### 2.1.1. Clinical Assessment and Scales

The panel of physicians emphasized that adult spasticity assessment should integrate descriptive, diagnostic, and prognostic tools, recognizing that neurological patients evolve over time. The Modified Ashworth Scale (MAS) remains useful for describing tone and facilitating interdisciplinary communication, particularly in early evaluations or hospital settings, but it should be complemented by functional and goal-oriented measures. The Goal Attainment Scaling (GAS) is preferred as the primary functional and prognostic tool because it structures objective-setting from the outset and captures individualized outcomes. In addition, the Barthel Index may be used to assess independence in activities of daily living, reflecting the patient’s functional status. In practice, the physicians recommended combining MAS and the Tardieu Scale for tone assessment, alongside GAS for functional tracking, to provide a comprehensive and clinically meaningful evaluation framework [8,9,10].

#### 2.1.2. Treatment Initiation

Treatment initiation should occur early to prevent progression of muscle overactivity. The panel recommends beginning botulinum toxin therapy, ideally within the first month after neurological injury or as soon as abnormal tone is detected, particularly in patients with marked paresis or sensory deficits, who are at higher risk of developing spasticity. BoNT-A should be regarded as first-line therapy in adults when muscle overactivity produces functional limitations [1,6,11].

A structured baseline evaluation was recommended as essential before the first injection and should document:

- Pain intensity and localization;
- Joint range of motion with measurable arcs;
- Tone severity;
- Functional capacity.

This anchors goal setting and allows objective assessment in subsequent cycles.

Initial treatment should begin with intermediate total doses to establish responsiveness and tolerability. Subsequent cycles should be goal-driven, adjusting dose magnitude, muscle selection, and distribution based on functional outcomes. The panel noted that injector experience influences dosing patterns, with less experienced injectors tending to use lower doses, while experienced injectors can safely administer intermediate-to-high doses when clinically appropriate.

Dosing must be individualized according to age, comorbidities, nutritional status, neurodegenerative conditions, and polypharmacy. In frail patients or those with impaired reinnervation, a conservative titration strategy is recommended. Ultimately, injections should be administered when selective muscle inhibition is expected to support a specific passive or active functional objective within the context of a coordinated rehabilitation program [4,6,10,11,12,13].

#### 2.1.3. Therapeutic Goals

Therapeutic goals should be defined at treatment initiation and aligned with the patient’s functional profile, clinical phenotype, and rehabilitation program. Realistic goal setting is essential to guide muscle selection, dose planning, and outcome evaluation and helps prevent dissatisfaction arising from unmet or misaligned expectations. GAS was identified as the preferred framework because it offers a structured, goal-oriented method to quantify clinically meaningful change over time [8].

The experienced injectors identified four common therapeutic domains:

- Pain and tone reduction: decreasing hypertonia and pain intensity, frequently among the earliest and most relevant benefits of BoNT-A.
- Range of motion and alignment: improving joint mobility and correcting abnormal postures or movement patterns, particularly in focal or segmental involvement.
- Functional optimization: enhancing gait, transfers, hygiene, self-care, and overall mobility, using functional scales to monitor progression.
- Integration into rehabilitation programs: ensuring that BoNT-A contributes meaningfully to the broader therapeutic plan, including physical therapy and adjunctive modalities when required.

#### 2.1.4. Muscle Selection

A detailed list of target muscles and pattern-based indications for adult spasticity is available in Appendix A Table A1.

#### 2.1.5. Recommended Technical Aids

The panel emphasized the need for accurate muscle localization. Anatomical landmark guidance is appropriate for superficial muscles, and the deepest and highest-risk muscles should be approached using dual guidance ultrasound and electromyography to ensure both anatomical precision and physiological confirmation.

#### 2.1.6. Practical Dose Ranges

The panel emphasized that maximum dosing in adult spasticity is highly variable and should reflect the severity of muscle overactivity, the number of segments involved, and the patient’s phenotype. Studies have reported safe use of BoNT-A doses above labeled limits [14].

The following practical dosage ranges and working dilutions were recommended:

- OnabotulinumtoxinA: 400–600 U (exceptional to 1000 U);
- IncobotulinumtoxinA: 600–800 U (exceptional to 1000 U);
- AbobotulinumtoxinA: up to 1500 U (exceptional cases to 2000 U).

Regulatory limits may be exceeded based on clinical experience or specific patient characteristics, but it must be clearly stated that this applies to exceptional cases.

Appendix A Table A2 and Table A3 presents recommended dosage ranges of each botulinum toxin type A suggested by the specialist group according to the selected muscle.

It is recommended to promote safety and efficacy studies in the Mexican population to define the use of high doses.

*Dilutions:

- OnabotulinumtoxinA 1–2 mL per 100 U;
- IncobotulinumtoxinA 1–2 mL per 100 U;
- AbobotulinumtoxinA ~2.5 mL per 500 U.

*The dilution varies according to the therapeutic goals and the target muscles [3,4].

#### 2.1.7. Re-Injection Interval

Re-injection intervals should reflect patient response, toxin characteristics, and functional objectives. Consistent with panel opinion:

- No adjustment doses should be given within 30 days, due to limited evidence and higher risk of adverse outcomes;
- The standard reinjection interval is 12 weeks for all formulations [3,11,15];
- A flexible 8–12 week interval is acceptable only for incobotulinumtoxinA, supported by published evidence originating from studies in blepharospasm and cervical dystonia and later extended to spasticity [3,15,16,17];
- In subsequent sessions, the number of treated muscles may be increased or decreased depending on the patient’s clinical evolution.

### 2.2. Pediatric Spasticity

#### 2.2.1. Clinical Assessment and Scales

Clinical assessment in pediatric spasticity must account for developmental variability, communication limitations, and the dynamic nature of tone abnormalities. According to the panel, MAS remains useful when other assessments cannot be performed, particularly in hospitalized, intubated, or low-responsiveness children. However, MAS should not be used as the sole determinant for treatment indication and should instead be complemented with functional and goal-oriented tools [9,18,19,20].

Gross Motor Function Measure (GMFM) is recommended for functional follow-up and GAS is the preferred method to document individualized progress, applicable across pediatric etiologies such as cerebral palsy and hereditary spastic paraplegia. Complementary scales such as the Tardieu Scale may assist in characterizing tone at specific joints [9,18].

#### 2.2.2. Treatment Initiation

The panel of clinicians recommended early identification of tone changes and timely initiation of injection therapy when abnormal muscle activity first becomes evident. In children at high neurological risk who develop spasticity that interferes with care and motor development, BoNT-A may be administered as early as 2 years of age, consistent with published pediatric safety data. The panel acknowledges that, in selected cases, BoNT-A has been safely administered below 2 years, based on experienced injector judgment and clear functional objectives. Early injections may be used not only to reduce spasticity but to enable developmental milestones, anticipating future functional goals [21,22,23].

The increase in tone must be evaluated within the broader context of central motor overactivity, not as an isolated phenomenon. Selective inhibition should only target muscles whose chemodenervation is expected to support meaningful functional outcomes, active or passive.

Pain-management strategies are frequently required in pediatric practices [24,25,26]. Physicians reported the use of:

- Inhaled sevoflurane for procedural anesthesia;
- Midazolam (oral);
- Hydroxyzine;
- Psychological techniques such as guided imagery and behavioral distraction.

#### 2.2.3. Therapeutic Goals

The treating physicians noted that therapeutic objectives in pediatric patients differ from adults by incorporating developmental milestones, preventing secondary musculoskeletal complications, and facilitating motor learning. Goals are typically organized into:

- Facilitation of emerging motor skills;
- Improvement of gait symmetry and efficiency;
- Optimization of passive function (hygiene, positioning, orthotic tolerance);
- Prevention of fixed contractures;
- Reduction of caregiver burden.

GAS is recommended as the primary framework for defining, documenting, and reassessing these goals [9].

#### 2.2.4. Muscle Selection

Muscle selection must reflect functional, growth trajectory, and expected developmental gains. Several key considerations were highlighted:

- Selection must follow clearly predefined goals.
- Children have variable muscle characteristics; US is strongly recommended for deep, small, atrophic, or surgically altered muscles.
- Avoid high-dose injections near the shoulder girdle due to the risk of respiratory complications.

#### 2.2.5. Recommended Technical Aids

US guidance is especially recommended for muscles such as the psoas, deep hip rotators, and tibialis posterior.

EMG or electrical stimulation when US cannot provide adequate discrimination of active fibers.

#### 2.2.6. Practical Dose Ranges

Dosing is strictly weight-based in pediatrics [27,28,29,30]. Recommendations include:

- OnabotulinumtoxinA: 8–15 U/kg total dose, not exceeding 400 U per session, with up to 600 U in adolescents approaching adult weight. Per-muscle guidance: 0.5–3 U/kg depending on severity, muscle size, and location.
- IncobotulinumtoxinA: 15–20 U/kg total dose, not exceeding 500 U per session. Per-muscle guidance: 1–4 U/kg depending on severity, muscle size, and location.
- AbobotulinumtoxinA: 10–30 U/kg total dose, up to 1000 U maximum. Per-muscle range: 1–8 U/kg, averaging at 3–6 U/kg depending on the muscle and phenotype.

Dose adjustments must consider muscle quality such as atrophy and altered viscoelastic properties, child’s nutritional condition, severity of spasticity, and anatomical regions where higher doses may increase the risk of adverse events.

Appendix A Table A4 and Table A5 present recommended dosage according to the selected muscle.

#### 2.2.7. Reinjection Interval

Injection intervals of at least three months are recommended and may be extended according to clinical response and patient needs, in alignment with established clinical experience and observed safety profiles [3,27,28,29,30].

### 2.3. Cervical Dystonia

#### 2.3.1. Clinical Assessment and Scales

Clinical assessment of cervical dystonia requires a precise and comprehensive characterization of the movement disorder. Evaluation begins with confirmation that the abnormal posturing is consistent with isolated cervical dystonia and not attributable to other conditions. The most frequent differential diagnoses of isolated cervical dystonia include alternative forms of cervical dystonia (such as cervical involvement in generalized dystonia, dystonia combined with additional movement disorders, paroxysmal dystonia, and dystonia associated with neurodegenerative diseases), as well as other conditions such as atlantoaxial subluxation, musculoskeletal disorders, cervical spine injury (traumatic, infectious, or neoplastic), and movement disorders including cervical motor tics and functional dystonia. All of these conditions may clinically overlap with dystonic postures or tremor-like presentations.

Accurate pattern classification is essential. Two complementary frameworks are typically applied: the classical nomenclature (torticollis, laterocollis, anterocollis, retrocollis) and the COL-CAP (Collis–Caput) system, which distinguishes neck-driven (collis) from head-driven (caput) components. Combining both systems allows for finer clinical resolution and more precise identification of the muscles contributing to the dystonic vector, particularly in mixed or complex presentations [31].

The Toronto Western Spasmodic Torticollis Rating Scale (TWSTRS-1) is the primary instrument used for multidimensional evaluation of severity, disability, and pain. The modified Tsui scale may serve as an alternative or complementary motor severity measure. When time allows and clinic workflow permits, quality-of-life-specific tools such as the Cervical Dystonia Impact Profile-58 may also be utilized [8,32,33].

It is also recommended evaluating non-motor symptoms, including anxiety, depression, pain fluctuations, sleep disturbances, and fatigue, as these manifestations are common in cervical dystonia and significantly impact treatment satisfaction and perceived therapeutic benefit.

#### 2.3.2. Treatment Initiation

Botulinum toxin type A should be initiated once a clearly defined dystonic pattern is established and results in pain, abnormal posture, functional limitation, or social impairment. The panel noted that treatment does not depend only on the degree of head deviation but on the identification of the dystonic driver and whether the pattern is tonic, phasic, or tremor-dominant. A baseline evaluation including pain scoring, range of motion, dystonic activation, and tremor characterization is recommended to guide target selection and to allow objective assessment in subsequent cycles.

Early treatment initiation was endorsed by the clinicians, as it may prevent maladaptive motor behavior and secondary musculoskeletal complications.

#### 2.3.3. Therapeutic Goals

Therapeutic goals center on restoring a more neutral and comfortable head posture, reducing pain, improving functional capacity in daily activities, and diminishing dystonic tremors when present. The panel stressed the importance of individualized goal setting based on the functional impact of the dystonic pattern. Clear communication of expected outcomes is essential to align patient expectations and facilitate consistent evaluation over time.

#### 2.3.4. Muscle Selection

The appropriate selection of muscles to inject requires a detailed analysis of the movement pattern exhibited by the cervical muscles, identifying the vectors of abnormal movement direction and selecting the muscles thought to contribute to that pattern. This can be subclassified based on the COL-CAP concept [34]. According to the displacement vectors, the most commonly injected muscles would be as follows:

Horizontal plane: cephalic rotation (torticaput)/cervical rotation (torticollis)

- Contralateral sternocleidomastoid, semispinalis capitis;
- Ipsilateral splenius capitis/cervicis, levator scapulae, inferior oblique of the head, trapezius.

Lateral plane: cephalic lateralization (laterocaput)/cervical lateralization (laterocollis)

- Ipsilateral sternocleidomastoid, levator scapulae, middle and posterior scalenes, trapezius, splenius capitis/cervicis, semispinalis capitis.

Anteroposterior plane: cephalic extension (retrocaput)/cervical extension (retrocollis)

- Bilateral splenius capitis/cervicis, semispinalis capitis/cervicis, trapezius, inferior oblique of the head.

Anteroposterior plane: cephalic flexion (anterocaput)/cervical flexion (anterocollis)

- Bilateral sternocleidomastoids, levator scapulae, anterior scalenes, longus colli/capitis.

Ultrasound guidance improves injection accuracy in the scalene muscles and deep cervical flexor and extensor muscles, while EMG guidance may assist in tremor-dominant patterns to identify active motor units [35,36].

#### 2.3.5. Recommended Technical Aids

Appendix A Table A6 shows a recommended guide for muscle localization.

#### 2.3.6. Practical Dose Ranges

Total dosing should be individualized according to pattern complexity, number of muscles involved, patient morphology, and response to previous treatment cycles. Higher doses are often required when both caput and collis components are active or when tremor is prominent. The physicians stressed caution when administering injections to deep anterior cervical muscles due to the elevated risk of dysphagia and airway complications.

The following practical working dilutions are recommended [3,27,29,30]:

- OnabotulinumtoxinA: 100 U/1 mL;
- IncobotulinumtoxinA: 100 U/1 mL;
- AbobotulinumtoxinA: 500 U/2.5 mL or 500 U/1 mL.

Instead of universal maximal values, dosing should reflect the distribution of dystonic activity across primary and accessory muscles.

Recommended dosage ranges will be provided in Appendix A Table A7.

#### 2.3.7. Reinjection Interval

The typical retreatment interval is approximately 12–16 weeks, corresponding to the expected duration of clinical benefit [3,26,28,29]. A flexible 8–12-week interval is acceptable only for incobotulinumtoxinA, supported by published evidence [3,15,16,17].

Some patients exhibit variable response duration depending on tremor predominance, muscle architecture, or cumulative treatment history. In subsequent applications, most physicians increase the dose and add new muscles according to the patient’s needs and response.

### 2.4. Management of Adverse Events in Adult and Pediatric Spasticity and Cervical Dystonia

The group of physicians agreed that adverse events associated with botulinum toxin type A in the management of adult spasticity are generally mild and transient, but emphasized that their anticipation and early control are essential. Local reactions such as pain, bruising, and swelling at the injection site are common and generally self-limited. More clinically significant events relate to excessive weakening of injected or adjacent muscles, which may manifest as impaired gait, diminished limb function, or, in cervical dystonia, reduced neck support or exaggerated compensatory postures. These events are usually reversible but indicate the need for dose reduction or improved muscle targeting in subsequent cycles.

Dysphagia is the most notable adverse event in cervical dystonia, arising particularly when deep or anterior cervical muscles are injected or when doses in these regions are too high. Management includes dietary adjustments, hydration support, and temporary referral to speech or swallow therapy. For subsequent injection cycles, lower doses or alternative muscle targets should be used, and injection technique should be optimized with US and EMG guidance.

In pediatric populations, the most common adverse events related to botulinum toxin administration include injection-site pain and muscle weakness. When injections involve cervical muscles or salivary glands, transient dysphagia may occur. In children with Gross Motor Function Classification System level V, respiratory difficulties have been reported; therefore, the use of maximum doses should be avoided. Focal cervical dystonia is not a common condition in the pediatric population, as it is usually part of a generalized or segmental dystonic syndrome, and no toxin has approval for use in dystonic syndromes in childhood.

Fatigue or flu-like symptoms may occur across all age groups and are generally self-limited, requiring only reassurance and symptomatic care.

A structured follow-up visit within two to four weeks allows clinicians to detect early complications, refine plans, and reinforce patient education regarding expected timelines and signs that require prompt attention. Early communication and proactive management help maintain treatment continuity, reduce discontinuation, and strengthen patient confidence.

All mitigation strategies and any dose escalation beyond local labeling should be individualized, explicitly documented, and implemented within a goal-based rehabilitation framework, weighing functional benefit against the risk of excessive weakness or systemic spread.

### 2.5. Management of Therapeutic Failure

Primary nonresponse (PNR) refers to a situation when BoNT-A fails to improve symptoms in patients [37]. The panel agrees that this situation is considered extraordinarily rare. When an apparent therapeutic failure is observed after an initial injection cycle, the first step is systematic clinical re-evaluation. In the case of spasticity, the group recommends reassessing the motor pattern, confirming the accuracy of the working diagnosis (spasticity vs. other forms of hypertonia or dystonia), revisiting the functional goals, and carefully reviewing the injection strategy. Often, a suboptimal response may be corrected by increasing the dose within safe limits, redistributing units among synergistic or antagonist muscles, or adding overlooked targets, while monitoring for weakness and functional impact.

For secondary nonresponse (SNR), defined as the loss of clinical benefit after an initial positive response to at least one injection [37], the group recommends a stepwise management approach for both spasticity and cervical dystonia management. This includes:

1. Comprehensive clinical reassessment and goal review;
2. Optimization of muscle selection and injection technique, preferably with guidance methods such as US, EMG, or electrical stimulation;
3. Consideration of laboratory evaluation for immune-mediated resistance when the pattern suggests neutralizing antibodies;
4. If resistance is documented, switching to a different BoNT-A formulation is advised;
5. Persistence of inadequate response despite a change in biologic may justify a washout period of approximately 8–12 months, while alternative interventions, including functional neurosurgical procedures, are considered, with BoNT-A retained as a potential adjunct for pain modulation and focal symptom control.

Despite generally favorable outcomes, treatment discontinuation rates vary, primarily due to cost, adverse effects, and a perceived lack of benefit, including indirect costs such as travel and limited access to care [37].

## 3. Discussion

The purpose of this article is to offer a practical, experience-based guide developed by a group of Mexican neurology and rehabilitation specialists who are highly experienced BoNT-A injectors and who routinely manage adult and pediatric spasticity as well as cervical dystonia. This document is not intended to replace the application guidance issued by pharmaceutical companies; rather, it aims to provide an updated, practice-oriented complement to existing treatment protocols within the Mexican context.

Across the three indications, our recommendations are largely consistent with international guidance in three core principles: (1) BoNT-A should be delivered within a goal-based, multidisciplinary rehabilitation model, (2) outcomes should be interpreted beyond tone reduction, prioritizing functional impact and goal attainment, and (3) injection accuracy and safety are improved by appropriate guidance techniques. These overarching positions align with major expert guidance for adult spasticity and consensus recommendations emphasizing structured assessment and integration with rehabilitation as well as implementation guidance [4,5,11].

Beyond assessment scales, dilution strategies, injection intervals, adjunctive techniques, and muscle selection, a distinctive contribution of this manuscript is the proposed dosing ranges for spasticity and cervical dystonia. These ranges are intended as practical reference points for experienced injectors rather than fixed prescriptions, and dose selection should remain individualized according to the clinical phenotype, treatment goals, previous response, and patient-specific risk profile. Taken together, these recommendations provide a structured basis for decisions that recur across successive BoNT-A treatment cycles, linking clinical assessment and goal setting with treatment planning, follow-up, and dose adjustment. This may help clinicians evaluate benefit in terms that are meaningful to the patient rather than relying solely on changes in muscle tone.

This framework may be particularly relevant in settings where access to specialized care, multidisciplinary rehabilitation, and injection-guidance technologies varies. Rather than proposing a uniform protocol, the recommendations allow core principles to be adapted to available resources and clinician expertise, while preserving product-specific prescribing information, regulatory requirements, and individualized clinical judgment.

In adult spasticity, the panel’s insistence that tone scales alone should not drive treatment decisions and that baseline evaluation should include pain, ROM, and functional capacity with explicit goals, closely matches guideline priorities. The Royal College of Physicians guideline supports goal-oriented planning and multidisciplinary follow-up [5], while international consensus/implementation documents, similarly stress structured goal-setting and reassessment to optimize outcomes [7,11].

Where these recommendations are more practice-oriented is in explicitly recognizing that certain complex phenotypes may require individualized dose escalation and broader muscle targeting, rather than a strictly label-driven approach, provided that safety monitoring prioritizes early detection of function-limiting weakness. This perspective is consistent with the clinical literature supporting high-dose use under expert monitoring in TOWER study and additional safety/practice analyses [14,15]. Importantly, these experience-based ranges should be interpreted as complementary to prescribing information, which remains the medico-legal safety baseline [27,29,30].

In pediatric spasticity, the emphasis on developmental context, individualized goals, and functional follow-up is consistent with pediatric reviews and expert recommendations that stress that pediatric BoNT-A should be framed around development and prevention of secondary complications, not tone reduction alone [22,23]. Our pragmatic view of MAS as a tool when cooperation is limited, while recommending complementary functional measures, aligns with the pediatric assessment literature, including reliability work for modified MAS and Tardieu scales [18]. A relevant nuance is the panel’s explicit attention to procedural comfort as a determinant of adherence and caregiver acceptance, which is increasingly supported by the evidence base in systematic review on pharmacologic sedation [25]. In dosing, our role remains aligned with weight-based and safety-focused approaches emphasized in reviews and consensus statements, like Russian consensus on pediatric incobotulinumtoxinA [22,28], while recognizing that local access and monitoring constraints may influence implementation.

For cervical dystonia, our diagnostic rigor (confirming isolated cervical dystonia and excluding mimics), structured phenotyping (including the COL-CAP system), and multidimensional measurement align with contemporary classification and expert recommendations [31,34]. For dosing and retreatment timing, our recommendations remain consistent with guideline-level evidence supporting BoNT in cervical dystonia in the American Academy of Neurology practice guideline [6] and with clinical trial/practice data allowing selected flexibility [17], while maintaining emphasis on dysphagia risk mitigation through careful target selection and dosing.

Guidance techniques are clinically relevant because they address two distinct sources of variability in BoNT-A treatment. Ultrasound provides real-time anatomical confirmation of muscle depth and borders, needle trajectory, and the relationship of the target muscle to adjacent neurovascular structures, whereas EMG provides physiological confirmation that the selected muscle contributes to the abnormal activation pattern. These modalities are therefore complementary rather than interchangeable. Their combined use may be particularly valuable when the clinical pattern is complex, the target muscle is deep or small, the anatomy is altered, or several adjacent muscles could plausibly contribute to the observed posture or movement. In these situations, guidance may help distinguish suboptimal muscle selection or needle placement from other potential causes of inadequate response, such as insufficient dosing or immunogenicity [3,11,35,36].

The practical role of each modality varies according to the indication and target muscle. In adult and pediatric spasticity, ultrasound is particularly useful for identifying deep or anatomically variable muscles and differentiating closely adjacent structures, while EMG or electrical stimulation may provide functional confirmation when selective muscle activation cannot be reliably determined through clinical examination alone. In cervical dystonia, ultrasound facilitates access to deep cervical muscles and visualization of nearby neurovascular structures, whereas EMG may help identify muscles with dystonic activity in complex or tremor-dominant patterns and differentiate primary dystonic drivers from compensatory activation. Nevertheless, US- or EMG-guided injection should not be considered mandatory for every muscle; its use should be individualized according to muscle depth, anatomical risk, pattern complexity, patient tolerance, available resources, and injector expertise [3,35,36].

Finally, our approach to insufficient response is consistent with expert algorithms across indications: before attributing failure to immunogenicity, clinicians should reassess diagnosis and pattern, revisit goals and expectations, and optimize targeting and technique, and then consider antibody evaluation and formulation switching when appropriate. This stepwise approach aligns with consensus guidance for botulinum toxin therapy [3] and immunogenicity reviews that emphasize technical and clinical drivers of apparent non-response, with neutralizing antibodies representing a narrower subset [37].

In summary, the panel’s recommendations align with international consensus and offer implementation-oriented guidance specific to Mexico. These practice-guidance recommendations reflect the contributions of experienced injectors, informed by a targeted evidence review and reported practice patterns. They are intended to support clinical decision-making rather than serve as prescriptive standards and may not fully capture all local resource constraints or rapidly emerging evidence.

### Limitations

This work has several limitations that should be considered when interpreting the recommendations. First, the supporting evidence was identified through a targeted narrative review rather than a systematic review, and no formal assessment of risk of bias or certainty of evidence was performed. Second, participants were selected through purposive sampling and were predominantly based in Mexico City; therefore, the recommendations may not fully reflect differences in resources, regulatory frameworks, and clinical practices across other regions. Third, the pre-meeting questionnaire relied on self-reported practice patterns, and the proposed dose ranges were derived from the values reported by experienced injectors rather than prospectively validated clinical outcomes. In addition, patients and caregivers were not directly involved, and their perspectives were not explicitly captured. Nevertheless, the structured questionnaire, nominal group technique, and integration of published evidence provided a systematic framework for developing practice-oriented recommendations. Accordingly, these recommendations should be interpreted as evidence-informed, experience-based guidance designed to support individualized clinical decision-making, rather than as prescriptive standards or conclusions about comparative effectiveness.

## 4. Conclusions

This document offers a comprehensive examination of the application of botulinum toxin in patients with spasticity, including both adults and children, as well as those with cervical dystonia. The information is derived from scientific literature and the experiences of a group of Mexican injectors who participated in a survey that addressed topics such as injected muscles and dosage ranges of botulinum toxin A.

It should be emphasized that the information presented herein must be considered in the context of the injector’s clinical expertise and the pursuit of individualized treatment goals. Moreover, this work aims to encourage experienced injectors to stay up to date by participating in courses and workshops.

## 5. Materials and Methods

### 5.1. Overall Approach and Design

This practice-guidance manuscript was developed through a structured three-phase process: (i) a targeted narrative literature review to identify relevant studies and guidance; (ii) a structured online survey to characterize current diagnostic and treatment practices; (iii) a face-to-face nominal group technique (NGT) meeting to refine practice statements.

### 5.2. Participants and Setting

Fifteen physicians participated, representing pediatric rehabilitation (n = 4), neurology (n = 4), physical and rehabilitation medicine (n = 3), neurorehabilitation (n = 3), and pediatric neurology (n = 1). Participants were based primarily in Mexico City with additional representation from other states of the republic. Practice settings included tertiary public hospitals like Instituto Mexicano del Seguro Social (Mexican Institute of Social Security, IMSS) and Instituto de Seguridad y Servicios Sociales de los Trabajadores del Estado (Institute of Security and Social Services for State Workers, ISSSTE), the Instituto Nacional de Neurología y Neurocirugía “Manuel Velasco Suárez” (National Institute of Neurology and Neurosurgery, INNN), a pediatric rehabilitation network (CRIT), an additional pediatric rehabilitation center, a regional high-specialty hospital, and private practice/other. Participants were purposively selected based on their extensive clinical experience with BoNT-A and routine management of spasticity and dystonia; most had more than 15 years of experience administering BoNT-A for these conditions.

### 5.3. Pre-Meeting Questionnaire

Two to three weeks before the face-to-face meeting, participants completed a structured online questionnaire to characterize baseline practice patterns and prioritize discussion topics. The questionnaire addressed: diagnostic approaches and outcome measures (tone, function, goal-based outcomes), muscle selection by clinical pattern, dilution practices, dose ranges and criteria for escalation, guidance modalities (anatomic palpation, US, electrical stimulation, EMG), reinjection intervals, adverse events and mitigation strategies, and approaches to inadequate response. The preliminary survey results were used to establish dose ranges; for each clinical scenario, the lower and upper bounds were defined by the minimum and maximum doses reported by participants.

### 5.4. Nominal Group Technique Procedures

NGT questions were organized into three indication-specific modules: (A) adult spasticity, (B) pediatric spasticity, and (C) cervical dystonia. Within each module, prompts were grouped across common domains: (1) evaluation and goal setting; (2) muscle selection and injection planning; (3) working dilutions and dosing ranges; (4) reinjection intervals and follow-up; (5) safety considerations; (6) management of inadequate response.

For each prompt, the NGT process comprised silent idea generation, round-robin sharing with real-time capture, and a structured clarification/consolidation phase. Draft statements were then prioritized and iteratively refined during moderated plenary discussion, with emphasis on actionability, safety, and feasibility in routine clinical practice. Statements were accepted at the end of each module by open agreement, defined as the absence of substantive unresolved objections.

### 5.5. Data Capture and Analysis

Meeting notes were summarized and coded using inductive thematic analysis to organize prioritized items into clinically meaningful domains within each indication. Reported ranges reflect real-world practice patterns captured in the preliminary questionnaire and should be interpreted alongside local labeling, regulatory guidance, and individual patient risk assessment. Discrepancies in thematic grouping and wording were resolved through iterative editorial review to ensure internal consistency across sections.

### 5.6. Targeted Narrative Literature Review

A targeted narrative review was performed to identify key clinical guidelines, society recommendations, and clinical studies relevant to BoNT-A use in adult spasticity, pediatric spasticity, and cervical dystonia. Searches were conducted in major biomedical databases (e.g., PubMed/MEDLINE, Scopus, Web of Science) using condition and intervention-related terms (e.g., spasticity, cerebral palsy, cervical dystonia, botulinum toxin, ultrasound guidance, electromyography, dosing, reinjection interval, immunogenicity). Evidence was used to support or contextualize prioritized items, particularly where dosing boundaries, guidance techniques, adverse-event prevention, and management of inadequate response were addressed.

### 5.7. Ethical Considerations

This work was a practice-guidance exercise and did not constitute a clinical research protocol. No patient-level data, biological samples, or clinical interventions were involved. The online pre-meeting questionnaire was completed voluntarily by healthcare professionals and captured only professional opinions, which were analyzed and reported in aggregated, anonymized form. Before participation, contributors were informed of the objectives, meeting procedures, and intended publication use of de-identified contributions, and provided informed agreement to participate. Accordingly, Institutional Review Board approval and patient informed consent were not required under applicable local regulations.

## Figures and Tables

**Table 1:** Summary of key practice statements.

Therapeutic Area	Key Practice Statements
**Adult spasticity**	- **Assessment:** Combine tone with function. MAS remains useful but should be complemented; GAS preferred for goal/outcome tracking; Tardieu for velocity-dependent components and hard-to-access muscles. - **Goals:** Define goals at initiation (aligned with phenotype/rehab). Core domains: pain/tone, ROM alignment, functional optimization and rehabilitation integration. - **Treatment initiation:** Start BoNT-A early, ideally within 1 month post-injury or once abnormal tone is detected. Document pain, ROM, tone severity, and functional capacity; MAS alone should not determine indication. - **Technique and dosing:** Use dual guidance (ultrasound + EMG) for deep or high-risk targets. Practical high-dose ranges and working dilutions are provided. Standard reinjection interval is 12 weeks; no “touch-up” injections within <30 days. Flexible 8–12-week intervals are acceptable only for incobotulinumtoxinA. - **Practical dose ranges:** OnabotulinumtoxinA 400–600 U; incobotulinumtoxinA 600–800 U; abobotulinumtoxinA up to 1500 U. - **Working dilutions:** OnabotulinumtoxinA 1–2 mL/100 U; incobotulinumtoxinA 1–2 mL/100 U; abobotulinumtoxinA ~2.5 mL/500 U. Dilution may vary by goals and target muscles.
**Pediatric spasticity**	- **Assessment**: Account for developmental variability. MAS may be used when other tools are not feasible, but should not be the sole determinant; GMFM is useful for functional follow-up; GAS is preferred for individualized progress; Tardieu may be used as an adjunct when needed. - **Goals:** Prioritize developmental milestones, prevention of secondary complications, and motor learning; GAS is recommended as the organizing framework. - **Treatment initiation**: initiate treatment early when abnormal activity emerges; procedural comfort often requires sedation/analgesia strategies. - **Technique and dosing:** Ultrasound guidance is strongly recommended for deep, small, atrophic or anatomically altered muscles. Avoid high doses near the shoulder girdle. Dosing is strictly weight-based with product-specific ranges; reinjection interval is approximately 3 months. - **Practical dose ranges:** OnabotulinumtoxinA 8–15 U/kg (≤400 U/session), incobotulinumtoxinA 15–20 U/kg (≤500 U/session), abobotulinumtoxinA 10–30 U/kg (≤1000 U/session).
**Cervical dystonia**	- **Assessment:** Confirm isolated cervical dystonia and exclude key differentials. Classify using classical nomenclature plus COL-CAP; TWSTRS primary scale. Evaluate relevant non-motor symptoms (mood, sleep, fatigue, pain fluctuations). - **Goals:** Focus on restoring a more neutral and comfortable head posture, reducing pain, improving daily functional capacity, and reducing tremor when present. Individualized goal setting and explicit expectation alignment are emphasized. - **Treatment initiation:** Initiate BoNT-A when a clearly defined dystonic pattern causes pain, abnormal posture, functional limitation, or social impairment. Treatment should be guided by the dystonic driver and by pattern type (tonic, phasic, or tremor-dominant), not only by degree of deviation. - **Technique and dosing:** Differentiate caput versus collis to define the dystonic vector. US improves accuracy in deep/small neck muscles; EMG may help in tremor-dominant patterns. Typical retreatment interval is 12–16 weeks; flexible 8–12-week intervals are acceptable only for incobotulinumtoxinA. Reinjection earlier than 12 weeks is not recommended without compelling indications. - **Practical dose ranges:** Total dosing should be individualized according to pattern complexity, number of muscles involved, patient morphology, and response to previous treatment cycles. Higher doses are often required when both caput and collis components are active or when tremor is prominent. - **Working dilutions:** OnabotulinumtoxinA 100 U/1 mL; incobotulinumtoxinA 100 U/1 mL; abobotulinumtoxinA 500 U/2.5 mL or 500 U/1 mL.

**Abbreviations:** **MAS:** Modified Ashworth Scale; **GAS:** Goal Attainment Scaling; **ROM:** range of motion; **GMFM:** Gross Motor Function Measure; **TWSTRS:** Toronto Western Spasmodic Torticollis Rating Scale; **COL-CAP:** Collis–Caput system.

## Data Availability

The original contributions presented in this study are included in the article. Further inquiries can be directed to the corresponding author.

## Appendix A

**Table A1:** Muscles targeted according to clinical presentation.

Region/Joint	Altered Clinical Pattern or Movement	Recommended Muscles	Experienced Injectors Recommendations
Shoulder/Arm	Elbow flexion, forearm pronation, internal rotation, and adduction	- Teres major	Use biceps only if the elbow is flexed and forearm is in supination. Teres major should be the primary target.
		- Biceps brachii (second line)	
Wrist	Palmar flexion and radial deviation	- Palmaris longus	Choose palmaris longus based on radial or ulnar deviation. Include FCR and/or FCU depending on the clinical case.
		- Flexor carpi radialis (FCR)	
		- Flexor carpi ulnaris (FCU)	
Hand and fingers	Digital flexion (superficial or deep)	- Flexor digitorum superficialis	Select based on spasticity severity and functional hand pattern.
		- Flexor digitorum profundus	
Hip	Adduction	- Pectineus	Apply based on clinical need.
Hip	Internal rotation and adduction	- Pectineus	Prioritize these muscles in this combined pattern.
		- Gracilis	
Hip	Isolated adduction	- Adductor longus	Main muscle for pure adduction.
Hip (flexors)	Flexion	- Iliacus	Differentiate application depending on clinical presentation.
		- Psoas major	
Knee	Persistent or incomplete flexion	- Knee flexor muscles (second line, as indicated)	Target as complementary muscles if therapeutic goals are not achieved.
Ankle	Forced inversion/altered contact phase	- Tibialis anterior	Consider application based on gait activity.

**Table A2:** Recommended dosage ranges of BoNT-A for adult upper limb muscles.

Pattern	Muscle	OnabotulinumtoxinA (U)	IncobotulinumtoxinA (U)	AbobotulinumtoxinA (U)
**SHOULDER**				
1. Adduction, elevation, flexion, and internal rotation	Pectoralis major	25–80	40–100	50–200
	Pectoralis minor	30–50	30–50	50–100
	Subscapularis	40–60	40–60	80–150
	Trapezius	25–75	25–75	50–150
	Levator scapulae	25–75	25–75	50–150
2. Abduction or adduction, extension, and internal rotation	Deltoid	20–50	20–50	30–100
	Pectoralis	25–75	25–80	50–150
	Latissimus dorsi	25–100	25–100	50–175
	Subscapularis	30–60	30–60	50–150
	Rhomboids	30–50	30–50	50–100
**ELBOW**				
1. Flexion. Forearm pronation	Biceps brachii	20–100	20–100	50–200
	Pronator teres	20–100	20–100	50–150
	Brachioradialis	20–100	20–100	50–200
2. Flexion. Forearm supination	Biceps brachii	20–150	25–150	50–200
	Brachialis	30–100	30–100	50–200
	Supinator	30–100	30–100	50–150
3. Flexion. Forearm in neutral position	Supinator	30–100	30–100	50–150
	Biceps brachii	25–100	25–100	50–200
4. Extension. Pronation	Triceps	25–150	25–150	50–300
	Pronator teres	30–100	30–100	50–150
**WRIST**				
1. Palmar flexión. Radial deviation	Palmaris longus	15–100	15–100	20–150
	Flexor carpi radialis	20–100	25–100	40–200
2. Palmar flexión. Ulnar deviation	Palmaris longus	15–100	15–100	30–150
	Flexor carpi ulnaris	15–100	15–100	30–150
3. Palmar flexión. Neutral	Palmaris longus	15–100	15–100	30–150
4. Palmar flexión. Extension	Wrist extensor	20–100	20–100	40–150
**HAND**				
1. Finger flexion—Deep predominance	Flexor digitorum superficialis	25–100	50–200	50–200
	Flexor digitorum profundus	25–100	25–100	50–200
	Lumbricals	10–50	10–50	15–100
	Palmar interossei	20–50	20–50	40–100
2. Thumb: Adduction, opposition, flexion, grasping	Flexor pollicis longus	10–50	10–50	20–150
	Flexor pollicis brevis	5–50	5–50	10–100
	Adductor pollicis	5–50	5–50	10–100
	Opponens pollicis	5–50	5–50	10–100

**Table A3:** Recommended dosage ranges of BoNT-A for adult lower limb muscles.

Pattern	Muscle	OnabotulinumtoxinA (U)	IncobotulinumtoxinA (U)	AbobotulinumtoxinA (U)
**HIP**				
1. Flexion, adduction, and internal rotation	Iliopsoas	40–150	40–150	50–300
	Adductor magnus	50–150	50–200	50–300
	Adductor longus	40–100	40–100	50–200
	Adductor brevis	30–100	30–100	50–150
2. Adduction and internal rotation	Adductor magnus	50–200	50–200	50–300
	Adductor longus	40–100	40–100	50–200
	Adductor brevis	30–100	30–100	50–150
	Gracilis	20–100	20–100	50–150
3. Extension and adduction	Gluteus maximus	50–100	50–100	50–250
	Adductor magnus	50–150	50–200	50–300
	Adductor brevis	30–100	30–100	50–150
**KNEE**				
1. Flexion	Biceps femoris	40–150	40–150	50–250
	Semitendinosus	30–100	30–200	50–200
	Semimembranosus	30–100	30–100	50–200
	Gracilis	50–100	50–100	50–250
2. Extension	Rectus femoris	40–100	40–100	50–200
**ANKLE**				
1. Plantar flexion and inversion	Gastrocnemius	25–100	25–100	50–250
	Soleus	30–100	30–100	50–250
	Tibialis posterior	25–100	25–100	50–250
	Tibialis anterior	25–100	25–100	50–200
	Flexor hallucis longus	20–75	20–75	40–150
**FOOT**				
1. Toe flexion	Flexor digitorum longus	25–75	20–75	50–200
	Flexor hallucis longus	20–75	20–75	50–150

**Table A4:** Recommended dosage ranges of BoNT-A for pediatric upper limb muscles.

Pattern	Muscle	OnabotulinumtoxinA (U)	IncobotulinumtoxinA (U)	AbobotulinumtoxinA (U)
**SHOULDER**				
1. Adduction, elevation, flexion, and internal rotation	Pectoralis major	5–75	5–100	10–200
	Pectoralis minor	20–40	20–40	30–70
	Subscapularis	5–50	5–60	10–80
	Trapezius	5–75	5–75	10–100
	Levator scapulae	25–75	25–75	50–150
2. Abduction or adduction, extension, and internal rotation	Deltoid	5–50	5–50	30–100
	Pectoralis	10–75	10–75	10–150
	Latissimus dorsi	10–100	10–100	10–150
	Subscapularis	5–50	5–60	10–80
	Rhomboids	30–50	30–50	50–75
**ELBOW**				
1. Flexion. Forearm pronation	Biceps brachii	20–100	20–100	50–150
	Pronator teres	10–50	10–50	10–100
	Brachioradialis	10–50	10–50	20–100
2. Flexion. Forearm supination	Biceps brachii	15–100	15–100	30–100
	Brachialis	10–50	10–50	10–100
	Supinator	15–50	15–50	30–100
3. Flexion. Forearm in neutral position	Supinator	15–50	15–50	30–100
	Biceps brachii	25–50	25–75	50–150
4. Extension. Pronation	Triceps	10–75	15–75	10–150
	Pronator teres	10–50	10–50	10–100
**WRIST**				
1. Palmar flexion. Radial deviation	Palmaris longus	10–50	10–50	20–100
	Flexor carpi radialis	10–100	10–100	20–150
2. Palmar flexion. Ulnar deviation	Palmaris longus	20–50	20–50	40–100
	Flexor carpi ulnaris	10–75	10–75	20–150
3. Palmar flexion. Neutral	Palmaris longus	30–50	20–50	40–100
4. Palmar flexion. Extension	Wrist extensor	10–50	10–50	10–100
**HAND**				
1. Finger flexion—Deep predominance	Flexor digitorum superficialis	10–50	10–50	20–100
	Flexor digitorum profundus	10–100	10–100	20–150
	Lumbricals	20–50	20–50	40–100
	Palmar interossei	10–75	10–75	20–150
2. Thumb: Adduction, opposition, flexion, grasping	Flexor pollicis longus	30–50	20–50	40–100
Pattern	Flexor pollicis brevis	10–50	10–50	10–100
	Adductor pollicis	10–50	10–50	20–100
	Opponens pollicis	10–100	10–100	20–150

**Table A5:** Recommended dosage ranges of BoNT-A for pediatric lower limb muscles.

Pattern	Muscle	OnabotulinumtoxinA (U)	IncobotulinumtoxinA (U)	AbobotulinumtoxinA (U)
**HIP**				
1. Flexion, adduction, and internal rotation	Iliopsoas	10–150	10–150	20–200
	Adductor magnus	5–200	5–200	10–250
	Adductor longus	5–75	5–75	10–100
	Adductor brevis	30–100	30–100	50–150
2. Adduction and internal rotation	Adductor magnus	15–100	15–100	30–150
	Adductor longus	5–200	5–200	10–250
	Adductor brevis	5–75	5–75	10–100
	Gracilis	5–50	5–50	10–85
3. Extension and adduction	Gluteus maximus	15–100	15–100	30–150
	Adductor magnus	30–150	30–150	50–200
	Adductor brevis	5–150	5–150	10–200
**KNEE**				
1. Flexion	Biceps femoris	20–150	20–150	40–200
	Semitendinosus	10–75	10–80	10–150
	Semimembranosus	10–75	10–80	10–150
	Gracilis	30–100	30–100	50–150
2. Extension	Rectus femoris	5–100	5–100	10–200
**ANKLE**				
1. Plantar flexion and inversion	Gastrocnemius	10–100	10–100	20–200
	Soleus	5–50	5–60	10–100
	Tibialis posterior	5–100	5–100	5–150
	Tibialis anterior	25–100	25–100	50–100
	Flexor hallucis longus	5–50	5–50	10–100
**FOOT**				
1. Toe flexion	Flexor digitorum longus	5–50	5–50	10–100
	Flexor hallucis longus	5–50	5–50	10–80

**Table A6:** Techniques recommended for muscle localization.

Anatomical Region	Muscles to Inject	Experienced Injectors Recommendations
Lateral surface	- Sternocleidomastoid	Direct anatomical application. Ultrasound guidance is recommended for deep muscles.
	- Scalenus medius	
	- Levator scapulae	
Posterior surface	- Trapezius	Standard anatomical approach. Consider muscle depth and fiber orientation.
	- Splenius capitis	
	- Semispinalis capitis	
Anterior surface	- Base of tongue muscles	Apply only when dystonic activity is identified. Use caution due to proximity to vascular/nervous structures.
	- Platysma	
Deep muscles, lateral surface	- Scalenus posterior	Ultrasound is recommended for precision and safety.
Deep muscles, posterior surface	- Splenius cervicis	Ultrasound guidance is advised and EMG is recommended when available. Blind injections should be avoided.
	- Semispinalis cervicis	
	- Obliquus capitis	
	- Rectus capitis	
Deep anterior surface (cervical region)	- Longus colli/Longus capitis	Dual guidance (ultrasound + EMG) is strongly recommended for optimal precision.

**Table A7:** Reference average BoNT-A dose in cervical dystonia.

Muscle	OnabotulinumtoxinA (U)	IncobotulinumtoxinA (U)	AbobotulinumtoxinA (U)
Sternocleidomastoids	20–75	20–100	50–150
Levator scapulae	15–75	15–75	25–200
Middle scalene	15–50	15–50	25–100
Posterior scalene	15–50	15–50	20–100
Trapezius	25–100	25–100	50–200
Splenius capitis	20–75	20–100	40–150
Splenius cervicis	15–80	15–80	25–200
Semispinalis capitis	15–80	15–100	25–200
Semispinalis cervicis	15–80	15–80	25–150
Longissimus capitis	15–50	15–75	25–100
Obliquus capitis inferior	10–50	10–50	20–150
Obliquus capitis superior	10–30	10–30	20–50
Rectus capitis major and minor	10–50	10–50	20–80
Anterior scalene	10–30	10–30	25–56
Longus colli	10–50	10–50	20–80
Submental complex	10–50	10–50	20–150
